# Treatment of HT29 Human Colorectal Cancer Cell Line with Nanocarrier-Encapsulated Camptothecin Reveals Histone Modifier Genes in the *Wnt* Signaling Pathway as Important Molecular Cues for Colon Cancer Targeting

**DOI:** 10.3390/ijms222212286

**Published:** 2021-11-13

**Authors:** Aisha Farhana, Avin Ee-Hwan Koh, Sangeetha Kothandan, Abdullah Alsrhani, Pooi Ling Mok, Suresh Kumar Subbiah

**Affiliations:** 1 Department of Clinical Laboratory Sciences, College of Applied Medical Sciences, Jouf University, Sakaka 72388, Saudi Arabia; afalserhani@ju.edu.sa (A.A.); rachelmok2005@gmail.com (P.L.M.); 2 Department of Biomedical Science, Faculty of Medicine and Health Sciences, Universiti Putra Malaysia, Serdang 43400, Malaysia; avin.keh@gmail.com; 3 Department of Biotechnology, Saveetha School of Engineering, SIMATS, Chennai 602105, India; sangeethak.sse@saveetha.com; 4 Department of Medical Microbiology, Universiti Putra Malaysia, Serdang 43400, Malaysia; 5 Centre for Materials Engineering and Regenerative Medicine, Bharath Institute of Higher Education and Research, Chennai 600126, India

**Keywords:** camptothecin, colorectal cancer, *Wnt* signaling pathway, bioinformatics, natural product

## Abstract

Cancer cells are able to proliferate in an unregulated manner. There are several mechanisms involved that propel such neoplastic transformations. One of these processes involves bypassing cell death through changes in gene expression and, consequently, cell growth. This involves a complex epigenetic interaction within the cell, which drives it towards oncogenic transformations. These epigenetic events augment cellular growth by potentially altering chromatin structures and influencing key gene expressions. Therapeutic mechanisms have been developed to combat this by taking advantage of the underlying oncogenic mechanisms through chemical modulation. Camptothecin (CPT) is an example of this type of drug. It is a selective topoisomerase I inhibitor that is effective against many cancers, such as colorectal cancer. Previously, we successfully formulated a magnetic nanocarrier-conjugated CPT with β-cyclodextrin and iron NPs (Fe_3_O_4_) cross-linked using EDTA (CPT-CEF). Compared to CPT alone, it boasts higher efficacy due to its selective targeting and increased solubility. In this study, we treated HT29 colon cancer cells with CPT-CEF and attempted to investigate the cytotoxic effects of the formulation through an epigenetic perspective. By using RNA-Seq, several differentially expressed genes were obtained (*p* < 0.05). Enrichr was then used for the over-representation analysis, and the genes were compared to the epigenetic roadmap and histone modification database. The results showed that the DEGs had a high correlation with epigenetic modifications involving histone H3 acetylation. Furthermore, a subset of these genes was shown to be associated with the *Wnt/β-catenin* signaling pathway, which is highly upregulated in a large number of cancer cells. These genes could be investigated as downstream therapeutic targets against the uncontrolled proliferation of cancer cells. Further interaction analysis of the identified genes with the key genes of the *Wnt/β-catenin* signaling pathway in colorectal cancer identified the direct interactors and a few transcription regulators. Further analysis in cBioPortal confirmed their genetic alterations and their distribution across patient samples. Thus, the findings of this study reveal that colorectal cancer could be reversed by treatment with the CPT-CEF nanoparticle-conjugated nanocarrier through an epigenetic mechanism.

## 1. Introduction

Colorectal cancer (CRC) is the third most common cancer reported globally. Genetic and epigenetic deregulation could lead to hyperactivation of the *Wnt* signaling pathway, which is prominently observed in this type of cancer. *Wnt* signaling regulates the maintenance of cancer cell stemness and metastasis [1].

Epigenetic modification is also one of the established drug resistance mechanisms encountered during the treatment process and possibly accounts for the failure of therapies for CRC [2]. In addition to the occurrence of aberrantly methylated genes, the miRNA level, chromatin remodeling, regulation by non-coding RNAs, genomic imprinting, and histone modifications (acetylation, methylation, and phosphorylation levels) are the other underlying mechanisms for the development of CRC [3,4,5]. These processes have been found to be reversible and are regulated through the epigenetic machinery of the cell. Thus, inhibiting the activity of histone-modifying enzymes, which can alter the cell’s epigenetic machinery, could be an effective tumor therapy approach.

Natural compounds also modulate epigenetic modifications by controlling DNA methylation and histone acetylation. The natural dietary compound folate, the vitamin genistein, catechols, curcumin, and raspberries have been demonstrated to influence the activity of DNMTs and to control DNA methylation and histone acetylation [6]. In specific, genistein has been shown to regulate the expression of genes in the *Wnt* signaling pathway in colon epithelial cells in rat models [7,8].

Camptothecin (CPT) has long been recognized as a potent anticancer drug, was approved by the United States Food and Drug Administration (FDA), and is being tested in human clinical trials [9]. CPT is a pentacyclic alkaloid that can be extracted from several types of plants, such as *Camptotheca acuminata*, *Chonemorpha grandiflora*, and *Nothapodytes nimmoniana*. It can form a ternary complex with DNA–Topoisomerase 1 to induce DNA damage by inhibiting the rejoining of a single-strand DNA break [10]. A DNA–Topoisomerase 1–CPT ternary complex has been used as the first line of drug treatment for cancer [11]. Presently, CPT, a Topoisomerase 1 inhibitor used against colorectal cancer, has been found to exhibit resistance, poor solubility, and increased toxicity, hence limiting its clinical applications [12]. To overcome these drawbacks, attempts have been made in the past decades to synthesize newer series of CPT derivatives and to improve their efficacy by conjugating them with nanoparticles [13].

Considering the importance of epigenetic mechanisms in colorectal cancer progression and the possibility of reversal through the application of appropriate drugs, we formulated CPT, a natural product with the help of nanocarriers, into a drug–nanoconjugate, the camptothecin β-cyclodextrin-EDTA-Fe_3_O_4_ nanoparticle-conjugated nanocarrier. We investigated the treatment effects of magnetic nanocarrier-conjugated CPT with β-cyclodextrin and iron nanoparticles (Fe_3_O_4_) cross-linked using EDTA (CPT-CEF) in the HT29 human colorectal carcinoma cell line. We further elucidated the gene expression profiling to identify epigenetically modified genes through over-representation analysis (ORA). We also checked the association of these epigenetically modified genes with the *Wnt* signaling genes. Further, the *Wnt* genes involved in colorectal cancer were studied for the interaction with the key epigenetically modified genes through protein–protein network analysis. The key genes identified by our analyses were correlated with the frequency of reports in colorectal cancer using cBioPortal.

## 2. Results

### 2.1. Identification of Genes Involved in Histone Modifications following Treatment with CPT-CEF in HT29 Colon Cancer Cells

**Figure 1:**
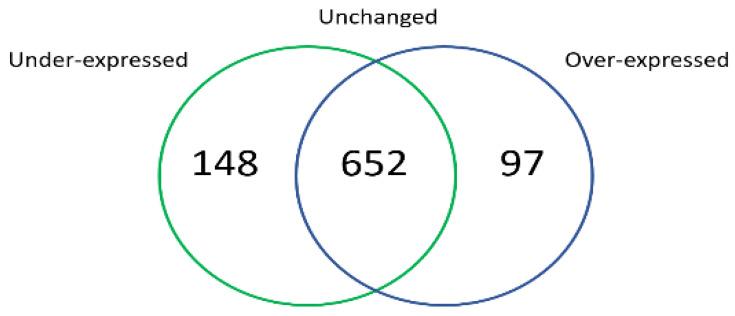
Venn diagram of differentially expressed genes between the CPT-CEF-treated and untreated HT29 colon cancer cells. The differentially expressed genes were calculated using DESeq2 and categorized based on underexpression, overexpression, and unchanged expression. The cut-off values of *p* < 0.1 and fold change > 2.0 (unchanged < 2.0) were used.

The differentially expressed gene profiles were determined with a threshold of two folds that was set for the CPT-CEF-treated and untreated HT29 cells. Genes showing an expression ratio greater than two folds were defined as differentially expressed genes (DEGs). Among the DEGs, 97 upregulated and 148 downregulated genes were identified (Figure 1).

**Figure 2:**
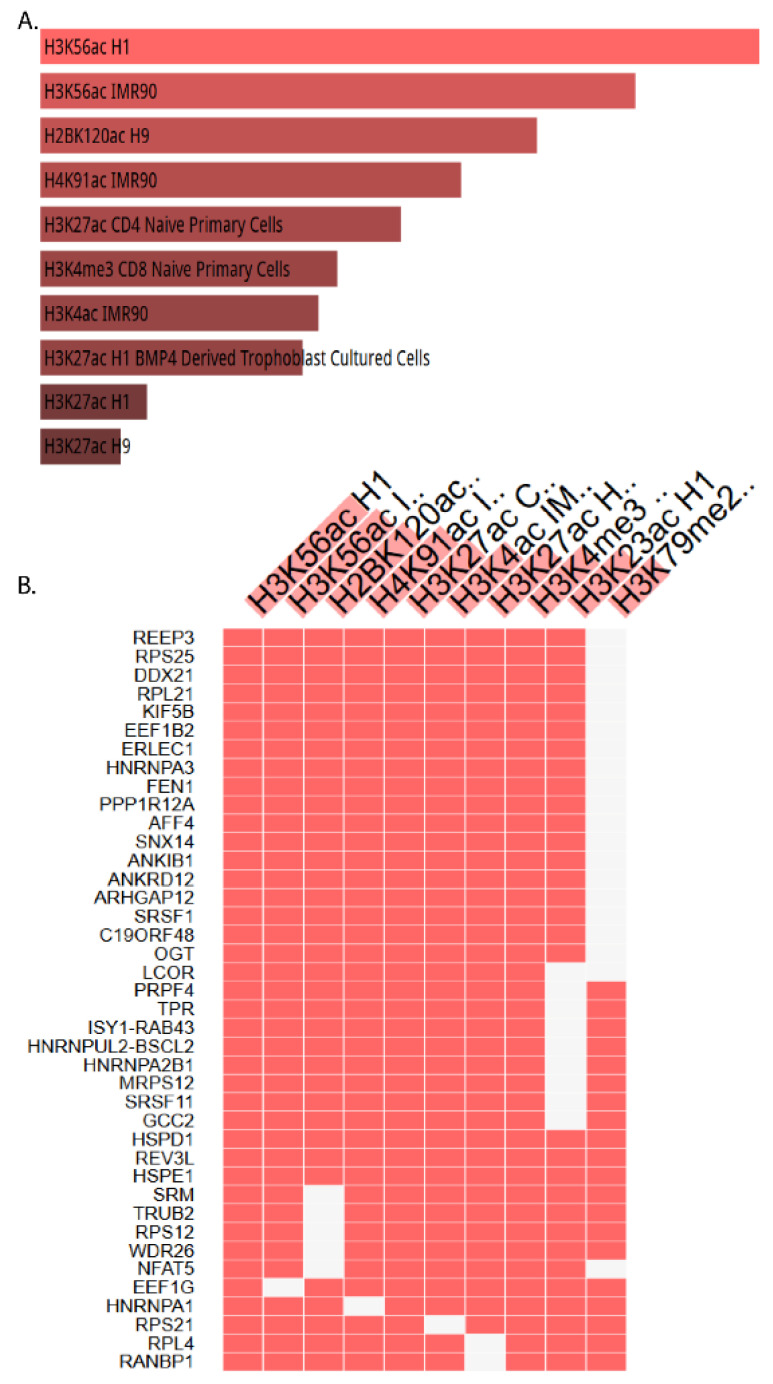
Visualization of the top 40 genes involved in histone modifications (**A**) and epigenomics (**B**) using over-representation analysis. The genes were obtained using ORA analysis, clustered by Enrichr for visualization (*p* < 0.05), and isolated according to the ENCODE Histone Modifications gene set library.

The thematic association of altered gene expression patterns to epigenetic mechanisms was uncovered through ORA using Enrichr. Both the upregulated and downregulated genes were used to search the Enrich database for the ENCODE Histone Modifications database. This provided information for identifying the associations with the genes involved in the histone modifications (Table 1). In total, 40 DEGs involved in histone modifications were identified through ORA. The computational search for histone modifications that could regulate the transcriptional patterns resulted in the identification of genes related to histone modifications at H3K27 (H3K27acL and H3K27acH), H3K4 (H3K4ac1M and H3K4mc3), H3K79 (H3K4me2), H3K56 (H3K56H1 and H3K56ac1.1), H2BK120ac 1, and H4K91ac 1 (Figure 2).

### 2.2. Identification of Epigenetic Modifier Genes Affecting the Wnt Signaling Pathway

**Figure 3:**
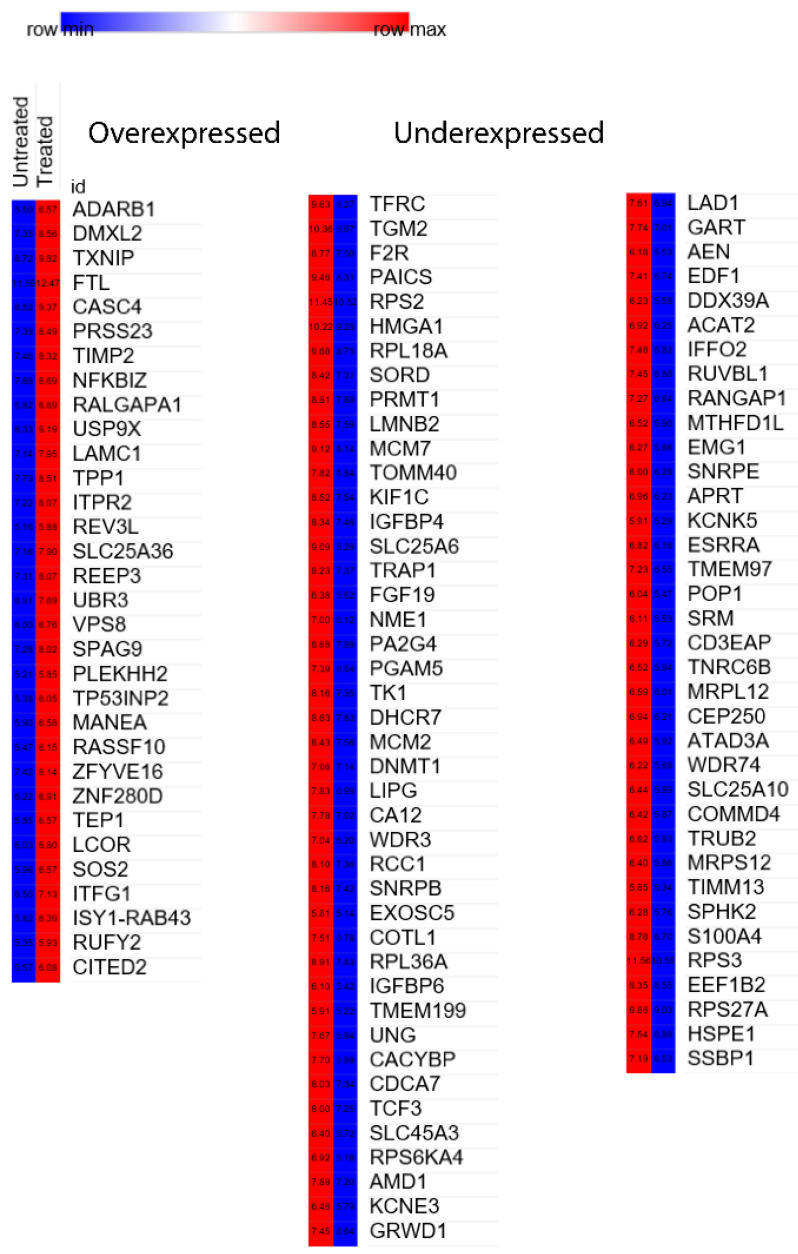
Heatmap of differentially expressed genes between the CPT-CEF-treated and untreated HT29 colon cancer cells. The differentially expressed genes involved in epigenetic mechanisms were measured using DESeq2 and categorized based on underexpression and overexpression (*p* < 0.05, fold change > ±2.0). Genes from the most over-represented H3K56ac epigenetic modifications were extracted and tabulated.

Figure 3 shows the genes involved in epigenetic mechanisms that are significantly altered upon treatment with CPT-CEF. Among the upregulated (overexpressed) genes, *REEP3*, *LCOR*, and *ISY1-RAB43* were found to be involved in histone modification, and the other genes were found to be downregulated (underexpressed) (Figure 3). Since genes involved in acetylation at H3K56 were most over-represented, the DEGs were further analyzed to derive sets of genes participating in the *Wnt* signaling pathways.

Further analysis that was performed to discern the associations of genes with the *Wnt* signaling pathway revealed a cluster of 29 genes with the involvement of nine upregulated genes and the remaining down-regulated genes (Table 2). The upregulated genes *ADARB1*, *TXNIP*, *FTL*, *TIMP2*, *USP9X*, *LAMC1*, *SLC25A36*, *TP531NP2*, and *RASSF10* exhibited a strong association with the *Wnt* signaling pathway. The functions for the upregulated genes associated with the *Wnt* signaling pathway were identified to be RNA binding, negative regulation of transcription by RNA polymerase II, iron ion binding, protease binding, being an extracellular matrix structural constituent, mitochondrial genome maintenance, autophagosome assembly, and spindle pole formation.

### 2.3. Protein–Protein Interaction (PPI) Networks and Functional Annotation

A total of 47 nodes and 160 edges were mapped in the PPI network with a local clustering coefficient of 0.572 and a PPI enrichment *p*-value of less than 1.0 × 10^−18^, with an average node degree of 6.81. The histone modifier genes that interacted with the *Wnt* signaling pathway gene are listed in Table 3, and among them, TCF7L2, TCF4, and SDC1 showed direct interactions with the MMP 7 gene, thus depicting their roles as direct activators in the pathway. Similarly C-myc interacted with SETD2, TMEM97, and H2AF7, whereas SMAD3 exhibited interactions with the USP9X and ZFYVE16 genes of the histone modification.

**Figure 4:**
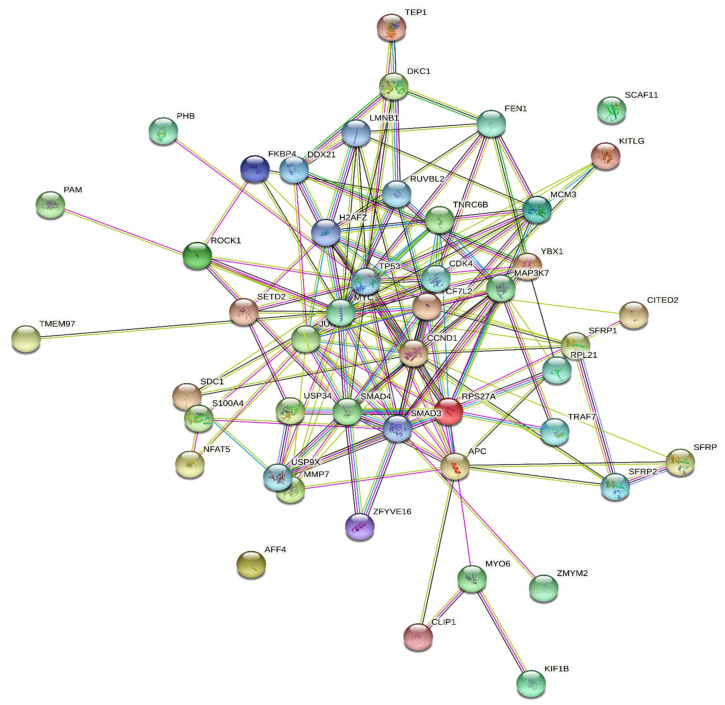
Protein–protein interaction networks. Each node in this protein–protein interaction network represents the single protein-coding gene locus, and the colored nodes are the first shell of interactors. Edges represent the protein–protein associations. The green color between the nodes represents the gene neighborhood.

MAPK37 showed an adjacent shell interaction with CF7L2, RPS27A, and RPL21; the C-Jun showed an interaction with the two key genes TMEM97 and SETD2, hence signifying that modifications or alterations in the interactors could directly influence the *Wnt* signaling pathway. Likewise, TP53 interacted with H2AFZ, TNRC6B, SETD2, SDC, and S100A4, while SSRP1 interacted with the AFFH genes. The CCND1 gene of the *Wnt* signaling pathway interacted with RPL21, RPS2AA, and SDC1 of the histone modifiers (Figure 4).

### 2.4. TCGA and Trans-Regulatory Elements

**Figure 5:**
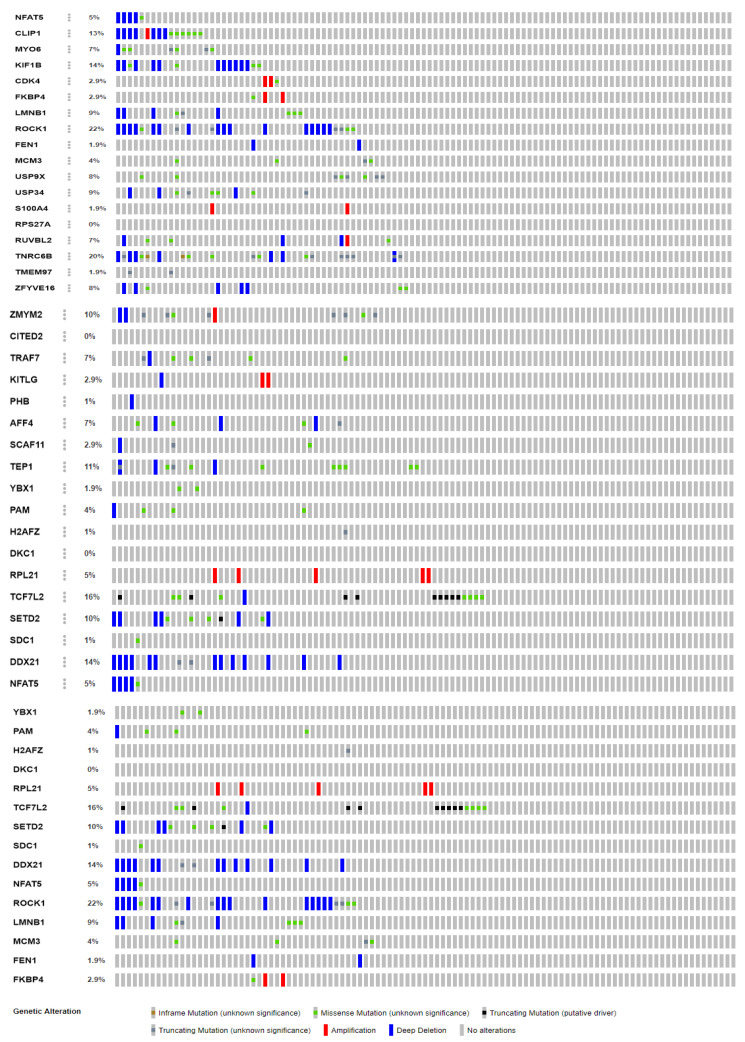
The frequency of reports of the key genes identified in colorectal cancer as analyzed in cBioPortal.

Further, the genes identified as direct interactors in *Wnt* signaling were analyzed for the metastatic occurrence percentage reported in metastatic colorectal cancer (MSKCC, Cancer Cell 2018, 1134 samples) in cBioPortal. The genes with the highest percentage were *CL1P1* (13%), *K1F1B* (14%), *ROCK1* (22%), *TNRC6B* (20%), *TCF7L2* (16%), *DDX21* (14%), and *LMNB1* (9%). However, the genes *CITED2* and *DKC1* were not reported in the patients. The distribution percentages of other genes and their specific genetic alterations resulting in colon cancer are represented in Figure 5. The interactions of the regulatory elements with the other transcription factors are presented in Table 4.

Further data mining for the trans-regulatory activities of the identified genes demonstrated that only *NFAT5* and *TCF7L2* acted as trans-regulatory elements, and the targets of *TCF7L2* were identified as *ABCB1*, *ACVRL1*, *CTNNB1*, *GATA3*, *GLCE*, *WWC1*, *PTGS2*, and *SNA12*. Specifically, *TCF7L2* acts as an activator of *BIRC5* and *STARD7* and the repressor of *HECA*. The other regulatory transcription factors identified were *AR*, *FOXA2*, *GATA3*, and *HNF4A*, while *TP53* acts as a repressor of *TCF7L2*. The targets of *NFAT5* were identified to be *CYP3A7*, *LTB*, and *TNF*, and these act as repressors of *NFAT5*.

## 3. Discussion

The significant challenges associated with colorectal cancer treatment are drug resistance and its side effects. The requirement of new drugs or modifications, specifically plant-based drugs, will undoubtedly serve as the solution. Patients’ demonstration of resistance to drug therapy, such as camptothecin therapy—even after one year of treatment—remains a challenge. Epigenetic modifications have been proposed to be a factor for drug resistance and colorectal cancer progression [1].

Hence, CPT-CEF nanoparticle-conjugated nanocarriers were synthesized and studied for their anticancer activities in the HT29 colon cancer cell line. Our previous study reported that following incubation with the CPT-CEF nanocompound solution, the cells exhibited a significant reduction in viability, disruption in the cell cycle, and changes in mitochondrial membrane potential. Cell death was confirmed with AO/PI double staining, and an increase in the release of caspase 3 into the supernatant was found (26). RNA sequencing was then undertaken to identify the differentially expressed transcripts after the treatment of drugs in the cells.

The ORA revealed the possible histone modification function of the 40 selected genes after the treatment of the HT29 colon cancer cells with the compound camptothecin with β-cyclodextrin-EDTA-Fe_3_O_4_ nanoparticle-conjugated nanocarriers.

In colorectal cancer, methylation of H3K4, H3K36, and H3K79 has been linked to the activation of gene expression, whereas H3K9me2, H3K9me3, H3K27me3, and H4K20 have been associated with gene repression [14,15,16]. Our computational search for histone modifications identified the interactions of differentially expressed genes at H3K4 (H3K4ac1M and H3K4mc3) and H3K79 (H3K4me2), possibly as histone acetylators, and they could thus be envisaged as epigenetic regulators. The role of H3K4me3 has also been implicated in transcriptional activation, along with histone H3 phosphorylated at tyrosine 41 (H3Y41). Several reports exist on the acetylation and the significant increase in colorectal cancer with the specific histone acetylators H3K27, H3K12ac, H3K18ac, H4K16, and H3Ac [17,18,19,20].

Notably, the upregulated and downregulated genes identified in this study are not directly correlated with colorectal cancer histone modification thus far. Hence, the identified genes could be investigated as potential epigenetically modulated therapeutic targets against uncontrolled proliferation of cancer cells.

Most genes work in an interrelated fashion, and thus a better evaluation of these genes in disease development could help in effective evaluation of treatment strategies. Identifying differentially expressed genes after drug treatment followed by over-representation analysis (ORA) and further association studies with the *Wnt* signaling pathway for possible histone acetylation genes revealed some interesting functional features. Subpathway analysis based on a signaling pathway impact analysis of the *Wnt* signaling pathway demonstrated both the canonical *Wnt/β-catenin* cascade and *Wnt*/planar cell polarity (*Wnt*/PCP) pathways were significantly enriched with DEGs relating to colorectal cancer [13].

We also identified a few specific subsets of genes involved in the *Wnt/β-catenin* signaling pathway and their functions. For instance, the TGM2 gene participates in blood vessel remodeling, DHCR7 in blood vessel development, S100A4 in the epithelial-to-mesenchymal transition, and F2R in the activation of MAPKK activity. Strikingly, the identified genes—namely, TXNIP, USP9X, DNMT1, and TCF3—could also be classified under the negative regulation of transcription by RNA polymerase II pathways. Hence, this study provides insights into the roles of differentially expressed genes as epigenetic regulators induced by CPT-CEF nanoparticle-conjugated nanocarriers and the involvement of a subset of genes in the *Wnt* signaling pathway.

To further confirm their interaction, protein–protein network analyses were carried out between the identified epigenetic modifiers and the 14 genes involved in the *Wnt*/*β-catenin* signaling pathway of colorectal cancer. Among the 345 genes, 47 interacted directly with the genes, thus implying the involvement of these 47 genes in the downregulation of the *Wnt*/*β-catenin* signaling pathway through protein–protein network analysis.

The deeper insights into the interactions of the key genes identified showed a strong connection with the *Wnt*/*β-catenin* signaling pathway associated with colorectal cancer.

Among the identified genes, a few were associated with pathways. *NFAT5*, a transcription regulator, regulates the *Wnt*/*β-catenin* signaling pathway and also influences the *S100A4* gene, which is a direct transcriptional target of the *Wnt/β-catenin/TCF*-mediated signaling pathway in colorectal cancer [21].

Interestingly, after drug treatment with camptothecin β-cyclodextrin-EDTA-Fe_3_O_4_ nanoparticle-conjugated nanocarriers, the *S100A4* gene was underexpressed. This implies the potential activity of the drug in the inhibition of colorectal cancer via the *Wnt/β-catenin/TCF*-mediated signaling pathway.

Study of other genes revealed that *LMNB2* showed an interaction with the *MYC* gene, and the overexpression of this nuclear protein was reported to inhibit colon cancer cell migration and interaction with chromatin [22].

The networking analysis showed the interaction of *CITED2* with *SMAD3*, which is involved in the *Wnt/β-catenin/TCF*-mediated signaling pathway. *CITED* has been reported to regulate colon cancer invasion directly and is considered as a target for HDAC-inhibitor-based intervention into colon cancer by butyrate, a naturally occurring HDAC inhibitor [23]. Similarly, *YB-1* and *TCF7L2* have been reported in association with colon cancer cells, wherein *YB-1* is involved in cell proliferation, migration, apoptosis, and EGFR expression in colorectal cancer [24], and *TCF7L2* is involved in the *Wnt/β-catenin* signaling pathway; polymorphism in that gene has been associated with increased risk of colon cancer [25]. In addition, it is important to report that the curation of the data did not result in the identification of a direct association of the remaining genes with colorectal cancer through the *Wnt* signaling pathway. Thus, among the 47 genes, the *NFAT5*, *S100A4*, *LMNB2*, *CITED2*, *YB-1*, and *TCF7L2* genes were identified as potential drug targets after the drug treatment in the HT29 cell lines, and they could also play a potential role in the epigenomics of colorectal cancer.

We further assessed the frequency of occurrence and the alterations of genes in colorectal patients to correlate the results obtained in our studies with the reported patient samples in order to gain more significance. The analysis in cBioPortal showed the significant distributions of the *CL1P1* (13%), *K1F1B* (14%), *ROCK1* (22%), *TNRC6B* (20%), *TCF7L2* (16%), *DDX21* (14%), and *LMNB1* (9%) genes. However, the *CITED2* and *DKC1* genes were not reported in the metastatic colorectal cancer samples (MSKCC, Cancer Cell 2018, 1134 samples). Among the alterations, most of the genes showed deep deletions, followed by amplification and truncated mutations.

In this study, a sequential bioinformatics analysis was carried out with the differentially expressed genes upon treatment with CPT-CEF nanoparticle-conjugated nanocarriers. The ORA was carried out for the genes involved in histone modifications and epigenomics. Further, the genes were searched for the involvement in the *Wnt* signaling pathway, and protein–protein interaction analysis was carried out for the genes involved in colorectal cancer. This study identified a few putative genes that directly contributed to the *Wnt* signaling pathways in colorectal cancer and two transcriptional regulators. Further analysis in cBioPortal confirmed their genetic alterations and their distribution in patient samples. Thus, this collective analysis revealed that epigenetically mediated colorectal cancer progression could possibly be reversed through the application of drug treatments with CPT-CEF nanoparticle-conjugated nanocarriers.

## 4. Materials and Methodology

### 4.1. Treatment of HT29 Colon Cancer Cells with a CPT-CEF Nanocompound

Before the experiment, a stock solution of the CPT-CEF nanocompound was formulated by dissolving the compound in 10% dimethyl sulfoxide (DMSO) (Naccalai, Kyoto, Japan) and complete culture medium. The HT29 human colorectal carcinoma cell line was acquired from the Laboratory of Vaccines and Immunotherapy (LIVES) in the Institute of Biosciences (IBS), UPM. Firstly, 1 × 10^4^ cells/mL were seeded in the culture medium in a 6-well culture plate. The culture media contained Roswell Park Memorial Institute (RPMI) 1640 medium (Naccalai, Kyoto, Japan), 10% fetal bovine serum (Naccalai, Kyoto, Japan), and 1% penicillin/streptomycin. The culture was then incubated at 37 °C in a 5% CO_2_ incubator for 24 h. After discarding the supernatant, the nanocompound solution was added to the culture medium to attain a final concentration of 133.5 μg/mL (IC_50_) (*n* = 3). The DMSO content in the stock solution did not exceed 1% of the final nanocompound concentration in the culture medium. The HT29 cell culture was then incubated for 48 h in a 5% CO_2_ incubator before the cells were harvested for RNA isolation for the RNA-Seq experiment.

### 4.2. Isolation of Total RNA from HT29 Colon Cancer Cells

The RNeasy mini kit was used to extract total RNA from CPT-CEF-treated and untreated HT29 colon cancer cells (*n* = 2) according to the manufacturer’s protocol (Qiagen, Hilden, Germany). In brief, 350 µL of RLT buffer was supplemented with 5% β-mercaptoethanol and then added to the cells for lysis. Homogenization of the samples was performed using a pipette. After adding an equal volume of 70% ethanol to the lysate, the mixture was aliquoted into the RNA spin column and centrifuged at maximum speed (12,000× *g*) for 15 s. After washing the column, the total RNA was eluted using 50 µL of RNase-free water. The purity and RNA integrity were then measured; for RNA integrity (RIN of at least 7), this was done by using the Bioanalyzer 2100 system (Agilent Technologies, Santa Clara, CA, USA), and for purity (A_260_/A_280_ ratio of approximately 2), it was done by using the NanoDrop 2000 Spectrophotometer (Thermo Scientific, Waltham, MA, USA).

### 4.3. Library Preparation for RNA-Seq

The NEBNext^®^ Ultra™ II RNA Library Prep Kit for Illumina^®^ (New England Biolabs, Ipswich, MA, USA) was used for the library preparation step according to the protocol mentioned in the handbook. Firstly, mRNA enrichment of total RNA was performed, followed by first-strand cDNA synthesis, as mentioned in the protocol. The next step was ligation of the adaptors to the library. Each ligation reaction mix contained a unique adaptor for the cDNA library. After that, the library was enriched with PCR and purified using the NEB Next Sample Purification Beads. The quality of the library was analyzed using the Bioanalyzer 2100 system. All of the samples showed a peak size of approximately 300 bp and were sufficient for RNA sequencing.

### 4.4. RNA-Seq Data Processing and Annotation

The sequencing platform used was the Illumina MiSeq system (Illumina, San Diego, CA, USA) with a read length of 2 × 100 bp. Approximately 4 million reads per sample were generated, with more than 80% of reads having good-quality scores (>Q30). The quality of the raw reads was then analyzed using FastQC. To map the RNA-Seq data to the human GRCh38 reference transcriptome (available at asia.ensembl.org, accessed on 18 July 2021) and perform quantification, the Salmon tool was used.

### 4.5. Differentially Expressed Genes between CPT-CEF-Treated and Untreated Colon Cancer Cells

The DeSeq2 tool (available at Bioconductor.org, accessed on 18 July 2021) was used to perform differential expression analysis. The reads were normalized based on each sample scaled by the medium-of-ratio method. A total of 11,118 transcripts were generated. By using adj *p* < 0.10 and a fold change of >2.0 as the threshold, 894 differentially expressed genes (DEGs) were isolated.

### 4.6. Over-Representation Analysis of Differentially Expressed Genes

The DEGs were profiled via over-representation analysis (ORA) by using Enrichr (https://maayanlab.cloud/Enrichr/, accessed on 18 July 2021). Homo sapiens was used as the reference. The g:SCS value was set as the significance threshold for multiple testing corrections, whereas the cut-off value was set to adj *p <* 0.05. The ENCODE Histone Modifications database in Enrichr was used to screen for epigenetic-related genes, and a heatmap was then constructed.

### 4.7. Construction and Analysis of Protein–Protein Interaction (PPI) Networks and Functional Annotation

The online tool STRING v11 (http://www.string-db.org, accessed on 18 July 2021) was used to construct an interactome map of the genes screened based on the ENCODE Histone Modifications database in Enrichr with the genes involved in the key *Wnt* signaling pathways. The PPI network was constructed via STRING with the default threshold of a combined score of more than 0.4, and then the PPI network was visualized with Cytoscape (version 3.6.1, NIGMS, NIH, USA). In addition, nodes represent biological molecules, and edges connect the nodes to indicate their relationship. The pivotal nodes in the PPI network were identified based on their connectivity degrees. The proteins that showed immediate interactions with the *Wnt* signaling genes associated with colorectal cancer were identified. The colorectal cancer genes *CTNBB1*, *APC*, *SMAD3*, *SMAD4*, *C-Myc*, *C-Jun*, *CCND1*, *TCF7L2*, *TP53*, *MMP7*, *MAP3K7*, *SFRP5*, *SFRP1*, and *SFRP2* were curated and subjected to the interactions.

### 4.8. TCGA Database and cBioPortal

The cBioPortal for Cancer Genomics (http://cbioportal.org, accessed on 18 July 2021) provides an open-access web resource for exploring, visualizing, and analyzing multidimensional cancer genomic data from TCGA. In the present study, three TCGA datasets of colorectal cancer—namely, metastatic colorectal cancer (MSKCC, Cancer Cell 2018, 1134 samples)—were selected to further analyze mutations, structural variants, and putative copy number alterations. The frequency of reports of the selected genes present in colorectal cancer was analyzed to correlate the significance of their interaction with the *Wnt* signaling pathway.

### 4.9. Identification of Transcription Factors and Their Interactions

The key genes identified from TCGA and cBioPortal were checked for their transcription regulatory roles using the TRRUSTV2 Database. TRRUST is the Transcriptional Regulatory Relationships Unraveled by Sentenced-based Text mining version 2.0 database (TRRUST), and it reveals the relationships between transcriptional regulations (http://www.grnpedia.org/trrust/, accessed on 18 July 2021) [26].

## 5. Conclusions

The differentially expressed genes identified in this study act as histone acetylators and exhibit a high correlation with epigenetic modifications in colorectal cancer. Furthermore, a subset of these genes was shown to be associated with the *Wnt/β-catenin* signaling pathway, which is highly upregulated in a large number of cancer cells. Further mechanistic studies and molecular pathways would help to evaluate them as potential downstream therapeutic targets. We propose that colorectal cancer could possibly be reversed by modulating these epigenetic mechanisms through CPT-CEF nanoparticle-conjugated nanocarriers.

## Figures and Tables

**Table 1:** A list of the top 10 terms was obtained using over-representation analysis via Enrichr and the ENCODE Histone Modifications gene set library.

Term	Gene Overlap	*p* Value	Adj *p* Value	Odds Ratio	Combined Score
H3K36me3 C2C12 mm9	43/407	4.64 × 10^−14^	1.91 × 10^−11^	3.827939	117.5226
H3K36me3 H7 hg19	106/1851	2.28 × 10^−13^	4.70 × 10^−11^	2.074867	60.39654
H3K79me2 myocyte mm9	107/2000	1.21 × 10^−11^	1.67 × 10^−9^	1.938406	48.72071
H3K36me3 bronchial epithelial cell hg19	92/1681	1.40 × 10^−10^	1.44 × 10^−8^	1.982947	44.9936
H3K36me3 spleen mm9	100/2000	2.71 × 10^−9^	2.23 × 10^−7^	1.811594	35.73849
H3K79me3 C2C12 mm9	97/2000	2.28 × 10^−8^	1.57 × 10^−6^	1.757246	30.91872
H3K36me3 Caco-2 hg19	31/382	9.48 × 10^−8^	5.58 × 10^−6^	2.940284	47.54937
H3K36me3 thymus mm9	66/1235	1.91 × 10^−7^	9.84 × 10^−6^	1.936279	29.95479
H3K36me3 MCF-7 hg19	72/1395	1.93 × 10^−7^	8.86 × 10^−6^	1.870033	28.90747
H3K36me3 ES-Bruce4 mm9	92/2000	6.19 × 10^−7^	2.55 × 10^−5^	1.666667	23.82415

**Table 2:** A list of the genes that were associated with *Wnt* signaling was obtained using over-representation analysis based on the ENCODE Histone Modifications gene set library from Enrichr.

Symbol	Function
ADARB1	RNA binding
TXNIP	negative regulation of transcription by RNA polymerase II
FTL	iron ion binding
TIMP2	protease binding
USP9X	negative regulation of transcription by RNA polymerase II
LAMC1	extracellular matrix structural constituent
SLC25A36	mitochondrial genome maintenance
TP53INP2	autophagosome assembly
RASSF10	spindle pole
TGM2	blood vessel remodeling
F2R	activation of MAPKK activity
HMGA1	DNA-binding transcription factor activity, RNA polymerase-II-specific
IGFBP4	regulation of cell growth
TRAP1	RNA binding
FGF19	MAPK cascade
NME1	magnesium ion binding
PGAM5	protein serine/threonine phosphatase activity
DHCR7	blood vessel development
MCM2	G1/S transition of mitotic cell cycle
DNMT1	negative regulation of transcription by RNA polymerase II
CACYBP	protein binding
TCF3	negative regulation of transcription by RNA polymerase II
RUVBL1	Swr1 complex
RANGAP1	Kinetochore
EMG1	blastocyst development
POP1	ribonuclease MRP activity
CEP250	G2/M transition of mitotic cell cycle
WDR74	nuclear exosome (RNase complex)
S100A4	epithelial to mesenchymal transition

**Table 3:** Interaction of the key genes of the *Wnt* signaling pathways of colorectal cancer with the DEGs identified as histone modifier genes.

Genes Associated with the *Wnt* Signaling Pathways of Colorectal Cancer	DEGs
*MMP7*	*NFAT5*, *CLIP1*, *MYO6*, *KIF1B*, *SETD2*, *SDC1*, *DDX21*, *NFAT5*, *TCF7L2*, *TCF4*, *SDC1*
*C-Myc*	*CDK4*, *FKBP4*, *LMNB1*, *ROCK1*, *FEN1*, *MCM3*, *SETD2*, *H2AF7USP9X*, *USP34*, *S100A4*, *RPS27A*, *RUVBL2*, *TNRC6B*, *TMEM97*, *KITLG*
*Smad3*	*ZFYVE16*, *ZMYM2*, *CITED2*, *USP9X*, *ZFYVE16*
*MAP3K7*	*TRAF7*, *RPL21*, *TCF7L2*, *CF7L2*, *RPS27A*, *RPL21*
*C-JUN*	*H24FZ*, *TMEM97*, *SETD2*
*TP53*	*PHB*, *TP53INF2*, *AC5L*, *H2AFZ*, *TNRC6B*, *SETD2*, *SDC*, *S100A4*
*SSRP1*	*AFF4*
*SRSF3*	*SCAF11*, *TEP1*, *EPR3A*, *YBX1*, *INRNPA3*
*CCND1*	*PAM*, *H2AFZ*, *RPL21*, *RPS27A*, *SDC1*
*SRSF5*	*DKC1*
*SRSF1*	*RSP10*
*SMAD*	*ROCK1*, *LMNB1*, *MCM3*, *FEN1*, *FKBP4*

**Table 4:** Interactions of the regulatory elements with the other transcription factors.

A	B	Neither	A Not B	B Not A	Both	Log2 Odds Ratio	*p*-Value	q-Value	Tendency
*TRAF7*	*SETD2*	1074	10	44	6	>3	<0.001	<0.001	Co-occurrence
*TCF7L2*	*SETD2*	782	97	29	12	1.738	0.002	0.005	Co-occurrence
*TRAF7*	*TCF7L2*	803	8	105	4	1.935	0.043	0.068	Co-occurrence
*CDK4*	*SETD2*	1078	6	48	2	2.904	0.045	0.068	Co-occurrence
*CDK4*	*TCF7L2*	805	6	108	1	0.313	0.588	0.705	Co-occurrence
*CDK4*	*TRAF7*	1110	8	16	0	<−3	0.892	0.892	Mutual exclusivity

## Data Availability

The data presented in this study are available on request from the corresponding author.
